# A Takagi–Taupin-Informed Simulation System and Multi-Parameter Optimization Method for Doubly Curved Crystal Monochromators in X-Ray Fluorescence Spectrometry

**DOI:** 10.3390/s26196071

**Published:** 2026-09-25

**Authors:** Zipeng Wang, Qi Zhang, Mohai Yue, Xiangjun Xin, Ran Gao, Lan Rao, Feng Tian, Yun Teng, Fu Wang

**Affiliations:** 1 School of Electronic Engineering, Beijing University of Posts and Telecommunications, Beijing 100876, China; 2 School of Information Engineering, Guangdong University of Technology, Guangzhou 510006, China; 3 School of Information and Electronics, Beijing Institute of Technology, Beijing 100081, China; 4 Beijing Ancoren Technology Co., Ltd., Beijing 101102, China

**Keywords:** X-ray fluorescence, doubly curved crystal, monochromator optimization, Takagi–Taupin theory, detection limit, Johann focusing, parameter scan

## Abstract

Doubly curved crystal (DCC) monochromators for X-ray fluorescence (XRF) couple crystal material, mosaicity, source/sample distances, crystal dimensions, and the meridional and sagittal bending radii, but are usually tuned empirically or one parameter at a time. A Takagi–Taupin-informed simulation system turns this coupled design problem into a common limit of detection (LOD) optimization by chaining tube spectrum modeling, an approximate diffraction module, two-dimensional DCC focusing geometry, and an XRF forward model from the source spectrum to the LOD. Calibrated using laboratory reference measurements and evaluated through a synthetic perturbation-based internal self-consistency test over 13 configurations, the system supports comparative screening within the explored design space; absolute LOD prediction still requires independent experimental validation. The engineering-recommended LiF(220) benchmark achieves an Fe Kα LOD of 1.253 ppm under shared geometry, while a separate Ge(111) curvature scan reaches a conditional sampled minimum of 1.094 ppm at $\left(R_{m},R_{s}\right)=\left(75,20\right)$ mm; the two values arise from different conditions and are not directly rankable. Curvature parameters dominate the LOD sensitivity, followed by the source distance, crystal dimensions, and anode selection. The framework is element-agnostic and transferable to other crystals and target elements.

## 1. Introduction

X-ray fluorescence (XRF) spectrometry exploits characteristic X-ray emission from inner-shell electronic transitions to perform rapid, non-destructive elemental analysis across a broad range of applications, including environmental monitoring [1], geological and extraterrestrial materials [2], plant science [3], biomedical imaging [4,5], bioanalytical chemistry [6], materials science, and cultural heritage conservation [7,8]. Recent reviews document sustained advances in XRF instrumentation, data processing, and multimodal imaging across laboratory, portable, and synchrotron platforms [9,10,11,12,13]. As trace element applications increasingly require ppm- or sub-ppm-level sensitivity under laboratory and compact instrument conditions, improving the excitation optics has become as important as detector-side signal processing. Detector resolution, scattering background, and matrix effects remain important general constraints in XRF, but the present work focuses specifically on the optical design problem of how to deliver a stronger and cleaner monochromatic excitation beam to the sample.

Wavelength-dispersive XRF (WD-XRF) employing flat or singly bent crystal analyzers provides a route to cleaner excitation optics by exploiting Bragg diffraction for narrow-band wavelength selection. Flat crystal geometries offer high energy resolution but collect only a small solid angle. Singly bent (meridionally curved) Johansson crystals focus radiation in the diffraction plane, improving throughput while maintaining wavelength selectivity [14,15,16]. Doubly curved crystal (DCC) monochromators extend this concept to two-dimensional focusing by adding a sagittal curvature $R_{s}$, thereby collecting a larger solid angle while maintaining spectral purity [14,15,16]. Beyond classical WD-XRF instrumentation, DCC-enabled monochromatic excitation has already supported compact laboratory and portable platforms for high-sensitivity XRF and related methods [16,17,18,19].

For hyperbolic DCC optics, however, the same two-dimensional focusing that improves collection also creates a difficult design problem. The meridional radius $R_{m}$ and sagittal radius $R_{s}$ do not simply maximize flux independently: they interact with the source distance, crystal dimensions, rocking curve width, Johann aberration, focal spot formation, and background acceptance. As a result, a DCC design with high integrated reflectivity or a large collected solid angle may still fail to produce the lowest detection limit if the bandwidth, spot shape, or peak-to-background ratio is unfavorable. These are optical design limitations rather than intrinsic limitations of XRF itself, and they are directly addressable by a model that propagates each DCC parameter to a common analytical metric.

Previous studies have demonstrated DCC-based XRF instrumentation, characterized the optical performance of curved crystals, or simulated individual diffraction and focusing properties [15,16,17,18,19,20,21]. These studies have established the feasibility and advantages of DCC-assisted monochromatic excitation. However, the principal optical quantities—including integrated reflectivity, diffraction bandwidth, collected solid angle, focal spot size, and peak-to-background ratio—are commonly evaluated separately. This limits their direct use in DCC design because improving one optical quantity does not necessarily improve analytical sensitivity. For example, broader angular acceptance may increase the delivered flux while simultaneously increasing spectral bandwidth and background, whereas a larger crystal aperture may improve collection but strengthen Johann aberration. A unified framework is therefore needed to propagate these competing optical effects to analyte signal, background, and LOD under common source, geometric, and engineering constraints.

The practical limitations to be resolved are thus concrete: selecting a crystal material and mosaicity without losing spectral purity, choosing $R_{m}$ and $R_{s}$ without confusing ideal geometric focusing with buildable curvature, setting source and sample distances under mechanical constraints, and comparing the tube material and operating power without separating them from the optical response. A useful design tool must connect these choices to the same terminal objective rather than optimizing the reflectivity, flux, or spot size in isolation.

To address this gap, the present work develops an analyte-oriented, end-to-end DCC design framework that propagates the incident tube spectrum through a Takagi–Taupin-informed diffraction surrogate, the meridional and sagittal focusing model, and an XRF forward model to a common LOD objective. The contribution is a unified DCC design methodology for coupled parameter screening rather than new diffraction theory. The main contributions are as follows:

(i) A parameterized approximate Takagi–Taupin-informed dynamical diffraction module with adaptive energy-grid sampling, anchored to reference integrated reflectivities and rocking curve widths.
(ii) An end-to-end XRF performance model linking the tube spectrum, DCC-transmitted excitation, sample fluorescence generation, background formation, and detector response.
(iii) A nine-anchor measurement-based calibration protocol constraining the diffraction, source, and bending modules, and a synthetic perturbation-based internal self-consistency check over 13 configurations (MRE =7.66%, r=0.9833, $k_{\mathrm{cal}}=1.026$).
(iv) Systematic multi-dimensional parameter scans that identify LiF(220) as the best common-geometry engineering benchmark with an Fe Kα LOD of 1.253 ppm, reveal a separate conditional sampled Ge(111) minimum of 1.094 ppm within the specified curvature scan at $\left(R_{m},R_{s}\right)=\left(75,20\right)$ mm, and show that curvature parameters dominate the local LOD sensitivity.

The originality of this work does not lie in introducing a new diffraction theory, but in integrating tube-spectrum generation, a Takagi–Taupin-informed diffraction surrogate, two-dimensional DCC focusing, and the XRF response into a unified LOD-based optimization framework. This integration enables coupled screening of crystal material, mosaicity, curvature, distances, dimensions, and source conditions, providing a practical design tool for laboratory and compact XRF instruments.

The remainder of the paper is organized as follows: Section 2 describes the theoretical framework. Section 3 integrates the simulation environment, parameter effect analysis, and DCC design optimization. Section 4 summarizes the main findings and concluding design implications.

## 2. Theoretical Framework

### 2.1. System Overview

The framework is implemented as a sequential forward model in which each module produces a limited set of outputs consumed by the next module:

(1) $$\Phi_{\mathrm{in}}\left(E\right)\to \left\{R_{\mathrm{int}},\Delta \theta_{\mathrm{eff}},\beta_{J}\right\}\to \left\{\Omega_{\mathrm{DCC}},\eta_{m},\eta_{s}\right\}\to \left\{I_{K\alpha },I_{\mathrm{bg}},\mathrm{LOD}\right\}.$$

The tube, diffraction, geometry, and XRF modules contribute these quantities in sequence.

Throughout the paper, the geometric parameters *p*, *q*, *L*, *W*, $R_{m}$, and $R_{s}$ are user inputs with millimeter units, and the crystal thickness $t_{c}$ is in micrometers; the crystal mosaicity η, the crystal material, and the anode material are material or source inputs; $\theta_{B}$ and the *d*-spacings, structure factors, attenuation coefficients, and fluorescence yields are database or standard physical values; and $k_{\mathrm{cal}}$, κ, $\beta_{J}$, $\eta_{m}$, and $\eta_{s}$ are calibrated or empirical parameters introduced below.

### 2.2. X-Ray Tube Spectrum

The bremsstrahlung continuum is modeled using the Kramers formula with the Kirkpatrick–Wiedmann (KW) correction [22,23]:

(2) $$\Phi_{\mathrm{brem}}\left(E\right)=K\cdot Z\cdot \frac{V_{0}-E}{E}\cdot f_{\mathrm{KW}}\left(E,Z\right),$$

where *K* is a scaling constant, *Z* is the anode atomic number, $V_{0}$ is the tube voltage (kV), *E* is the photon energy (keV), and $f_{\mathrm{KW}}$ is the dimensionless KW correction factor. Characteristic lines are added as Pseudo-Voigt profiles:

(3) $$\Phi_{\mathrm{char}}\left(E\right)=\sum_{i}A_{i}\cdot \mathrm{PV}\left(E-E_{i},\;\Gamma_{i},\;m_{i}\right),$$

where $A_{i}$ is the amplitude, $E_{i}$ the centroid energy (keV), $\Gamma_{i}$ the line width (keV), and $m_{i}$ the dimensionless Pseudo-Voigt mixing fraction of characteristic line *i*.

Transmission through the beryllium exit window is applied as

(4) $$T_{\mathrm{Be}}\left(E\right)=\exp\;\left(-\mu_{\mathrm{Be}}\left(E\right)\;\rho_{\mathrm{Be}}\;t_{\mathrm{Be}}\right).$$

where $T_{\mathrm{Be}}\left(E\right)$ is the beryllium window transmission, $\mu_{\mathrm{Be}}\left(E\right)$ the mass attenuation coefficient of beryllium (cm^2^/g), $\rho_{\mathrm{Be}}$ the beryllium density, and $t_{\mathrm{Be}}$ the window thickness; the product $\mu_{\mathrm{Be}}\rho_{\mathrm{Be}}t_{\mathrm{Be}}$ is dimensionless.

The modeled source fluxes integrated above the Fe K-edge at 50 kV/40 mA are $1.398\times 10^{17}$, $5.318\times 10^{16}$, and $1.267\times 10^{17}$ ph/s for Ag, Cr, and Mo, respectively, yielding an Ag/Cr integrated source flux ratio of 2.63× against the measured reference anchor of 3.20×. The tube spectrum module thus delivers the incident spectral flux $\Phi_{\mathrm{in}}\left(E\right)$ as input to the crystal diffraction module. The overall workflow of the proposed simulation framework is shown in Figure 1.

### 2.3. Approximate Takagi–Taupin-Informed Diffraction Module

The approximate dynamical diffraction module is used as a computationally efficient surrogate rather than a full numerical solution of the bent crystal Takagi–Taupin equations. Its internal data flow is

(5) $$F_{h}\left(E\right)\to \left\{\Delta \theta_{\mathrm{Darwin}}\left(E\right),\Lambda_{\mathrm{ext}}\left(E\right)\right\}\to R\left(E\right)\to \left\{R_{\mathrm{int}},\Delta \theta_{\mathrm{eff}},\beta_{J}\right\}.$$

Stage 1 computes the structure factor, Stage 2 converts $|F_{h}\left(E\right)|$ into the Darwin width and extinction length, Stage 3 estimates the peak reflectivity from the normalized thickness $\xi =t_{c}/\Lambda_{\mathrm{ext}}$, and Stage 4 applies mosaic and Johann broadening to produce the diffraction outputs used downstream.

The first step takes the crystal basis, reflection vector, and photon energy as inputs and computes the energy-dependent structure factor [24]:

(6) $$F_{h}\left(E\right)=\sum_{j}\left[f_{j}^{0}\;\left(\frac{\sin\theta }{\lambda }\right)+f_{j}^{\prime }\left(E\right)+if_{j}^{\prime \prime }\left(E\right)\right]e^{-B_{j}\sin^{2}\theta /\lambda^{2}}\;e^{2\pi i\;h\cdot r_{j}},$$

where $B_{j}$ is the Debye–Waller factor, $r_{j}$ the atomic position, and λ the X-ray wavelength. The resulting $|F_{h}\left(E\right)|$ sets the amplitude scale of the Darwin width and extinction length.

The intrinsic angular and length scales of the perfect-crystal response are then estimated using [25,26,27]

(7) $$\Delta \theta_{\mathrm{Darwin}}\left(E\right)=\frac{2r_{e}\lambda^{2}\left|F_{h}\right|}{\pi V_{c}\sin2\theta_{B}},\;\Lambda_{\mathrm{ext}}\left(E\right)=\frac{\pi V_{c}\cos\theta_{B}}{r_{e}\lambda \left|F_{h}\right|},$$

where $r_{e}$ is the classical electron radius (in m), $V_{c}$ the unit-cell volume (in m^3^), $\theta_{B}$ the Bragg angle, and $t_{c}$ the crystal thickness (in micrometers). The Darwin width $\Delta \theta_{\mathrm{Darwin}}$ is later combined with mosaic and Johann broadening, whereas the extinction length $\Lambda_{\mathrm{ext}}$ is used to form the normalized thickness $\xi =t_{c}/\Lambda_{\mathrm{ext}}$.

The peak reflectivity is then approximated by a compact piecewise surrogate that connects the kinematical and dynamical limits [25,26,28]:

(8) $$R\left(E\right)=\left\{\begin{array}{cc} {\left(\frac{\pi \xi }{2}\right)}^{2} & \xi \ll 1\;\left(\mathrm{kinematical}\right),\\ \tanh^{2}\left(\xi \right) & \xi \gg 1\;\left(\mathrm{dynamical}\right). \end{array}\right.$$

For ξ≈1, a smooth interpolation between the two asymptotic limits is used.

The output of this stage is the energy-resolved peak reflectivity R(E), the single quantity carried forward into the mosaic and Johann convolution. For asymmetric cuts with asymmetry angle α, R(E) is modified by the asymmetry factor $b=\sin\left(\theta_{B}-\alpha \right)/\sin\left(\theta_{B}+\alpha \right)$ to give $R_{\mathrm{asym}}\left(E\right)=\sqrt{b}\;R\left(E\right)$ before being passed downstream.

Finally, the reflectivity response is converted into the diffraction quantities required by the geometry and XRF modules. For mosaic crystals,

(9) $$R_{\mathrm{int}}=\int R\left(\Delta \theta \right)\;G\left(\Delta \theta ;\eta \right)\;d\left(\Delta \theta \right),$$

where Δθ is the angular offset from the Bragg angle; G(Δθ;η) is a Gaussian mosaic distribution with FWHM equal to the mosaicity η (in degrees), a crystal input parameter; and $R_{\mathrm{int}}$ is the integrated reflectivity. The effective rocking curve width combines three contributions of distinct physical origin: the Darwin width inherited from Stage 2, the mosaicity that characterizes the crystal itself, and a Johann aberration term whose magnitude is set by the bending geometry developed in Section 2.4. The three terms are combined as a quadrature sum under the approximation that they act as independent broadening mechanisms, and the Johann term is a geometric estimate rather than a strictly Gaussian convolution:

(10) $$\Delta \theta_{\mathrm{eff}}=\sqrt{\eta^{2}+\Delta \theta_{\mathrm{Darwin}}^{2}+\delta_{\mathrm{Johann}}^{2}}\;,\;\delta_{\mathrm{Johann}}=\frac{L^{2}}{8R_{m}^{2}\tan\theta_{B}}.$$

All widths are converted to radians and expressed as Gaussian-equivalent FWHMs. Under the independent Gaussian-equivalent approximation, the quadrature rule follows from variance addition.

The Johann term introduces the geometry variables *L* and $R_{m}$ into the diffraction response. The corresponding background acceptance penalty is treated as a calibrated phenomenological background-scaling factor rather than a universal physical law:

(11) $$\beta_{J}={\left(\frac{\Delta \theta_{\mathrm{eff}}}{\sqrt{\eta^{2}+\Delta \theta_{\mathrm{Darwin}}^{2}}}\right)}^{\;2}.$$

The squared ratio approximates the increase in the two-dimensional angular acceptance area and is normalized to $\beta_{J}=1$ in the absence of Johann broadening.

The outputs $R_{\mathrm{int}}$, $\Delta \theta_{\mathrm{eff}}$, and $\beta_{J}$ are passed to Section 2.4 and Section 2.5 to determine the collected flux and background.

### 2.4. DCC Focusing Geometry

The geometry module converts the diffraction acceptance from Section 2.3 into the collection efficiency and focal spot gain. It takes *p* (source-to-crystal distance), *q* (crystal-to-sample distance), *L* (meridional crystal length), *W* (sagittal crystal width), $R_{m}$ (meridional bending radius), and $R_{s}$ (sagittal bending radius) as geometric inputs, all in millimeters, and returns $\Omega_{\mathrm{DCC}}$, $\eta_{m}\left(R_{m}\right)$, and $\eta_{s}\left(R_{s}\right)$ for the XRF forward model to use.

The ideal meridional Rowland condition gives

(12) $$R_{m}^{\mathrm{opt}}=\frac{2p}{\sin\theta_{B}},$$

and the ideal sagittal focusing condition gives

(13) $$R_{s}^{\mathrm{opt}}=\frac{2pq}{p+q}\;\sin\theta_{B},$$

where $\theta_{B}$ is inherited from the crystal *d*-spacing and target excitation energy. The ideal radii are used as geometric references, while compact laboratory configurations are represented through semi-empirical reduced-order focusing factors.

For meridional bending, the practical efficiency is modeled as

(14) $$\eta_{m}\left(R_{m}\right)=\exp\;\left[-\frac{1}{2}{\left(\frac{\ln(R_{m}/R_{m}^{\mathrm{ref}})}{\sigma_{m}}\right)}^{\;2}\right],\;R_{m}^{\mathrm{ref}}=85\;\mathrm{mm}\cdot \frac{p}{150\;\mathrm{mm}},$$

where $R_{m}^{\mathrm{ref}}$ is the reference meridional radius and $\sigma_{m}=0.60$.

Motivated by the curvature-dependent focusing behavior of doubly bent crystals [28], the log-normal form provides a positive and smooth response to any relative meridional curvature mismatch, while $\sigma_{m}=0.60$ is an empirically calibrated dimensionless curvature-tolerance parameter rather than a universal crystal constant. The single bending uniformity anchor at $R_{m}=85$ mm constrains the model at one radius but does not validate the full functional dependence of $\eta_{m}$.

For sagittal focusing,

(15) $$\eta_{s}\left(R_{s}\right)=\frac{f_{\mathrm{spot},\mathrm{opt}}}{f_{\mathrm{spot}}\left(R_{s}\right)}\leq 1,$$

The factor $\eta_{s}$ is defined as the ratio of the optimal spot width to the modeled spot width and approximately describes the photon concentration loss induced by the sagittal mismatch, where $f_{\mathrm{spot}}\left(R_{s}\right)$ is the modeled focal spot width (in µm) and accumulates contributions from source demagnification, defocus due to $R_{s}\neq R_{s}^{\mathrm{opt}}$, and Darwin-width broadening.

The two semi-empirical reduced-order dimensionless focusing factors $\eta_{m}$ and $\eta_{s}$ jointly scale the reflected flux delivered to the sample and are intended for comparative screening and engineering design. The focusing geometry and focal spot in Figure 2 are model-generated.

The aperture-dependent collection term is represented by

(16) $$\Omega_{\mathrm{DCC}}=\frac{L\cdot W}{p^{2}},$$

where L×W is the active crystal aperture and *p* is the source-to-crystal distance such that $\Omega_{\mathrm{DCC}}$ is a dimensionless solid angle (sr) in the small-angle approximation. The geometric gain over a single-bend reference is

(17) $$G=\frac{\Omega_{\mathrm{DCC}}}{\Omega_{\mathrm{SB}}}=\frac{W}{L\;\Delta \theta_{\mathrm{eff}}}.$$

Section 2.4 thus provides $\Omega_{\mathrm{DCC}}$, $\eta_{m}$, and $\eta_{s}$ to the XRF module, while $\Delta \theta_{\mathrm{eff}}$ from Section 2.3 enters the gain and background terms.

### 2.5. XRF Forward Model and Detection Limit

The net fluorescence signal intensity follows the standard fundamental parameters treatment of XRF with self-absorption correction [29,30,31,32]:

(18) $$I_{K\alpha }=\Phi_{\mathrm{out}}\cdot \frac{\mu_{\mathrm{pe}}\left(E_{0}\right)}{\mu_{\mathrm{tot}}\left(E_{0}\right)+\mu_{\mathrm{tot}}\left(E_{K\alpha }\right)\csc\psi }\cdot \omega_{K}\cdot C\cdot \epsilon_{\det}\cdot \Omega_{\det},$$

where $I_{K\alpha }$ is the net fluorescence count rate (counts/s), $\Phi_{\mathrm{out}}$ is the output flux delivered to the sample (photons/s), $E_{0}$ the incident excitation energy (keV), $E_{K\alpha }$ the fluorescence line energy (keV), $\mu_{\mathrm{pe}}$ and $\mu_{\mathrm{tot}}$ are the photoelectric and total mass attenuation coefficients (cm^2^/g), ψ is the take-off angle (in degrees), $\omega_{K}$ is the fluorescence yield (dimensionless), *C* is the elemental concentration, $\epsilon_{\det}$ is the detector efficiency (dimensionless), and $\Omega_{\det}$ is the detector solid angle (sr).

The background level is

(19) $$I_{\mathrm{bg}}=\frac{\Phi_{\mathrm{out}}\cdot \kappa }{{\left(P/B\right)}_{\mathrm{crystal}}}\cdot \beta_{J},$$

where $I_{\mathrm{bg}}$ is the background count rate (counts/s), $\kappa =3.5\times 10^{-8}$ is a dimensionless calibrated background coefficient, ${\left(P/B\right)}_{\mathrm{crystal}}$ is the dimensionless crystal peak-to-background ratio (P/B), and $\beta_{J}\geq 1$ is the dimensionless Johann broadening penalty from Section 2.3.

The detection limit follows the Currie criterion [33]:

(20) $$\mathrm{LOD}=\frac{3\sqrt{I_{\mathrm{bg}}}}{S\sqrt{t}}\cdot k_{\mathrm{cal}},$$

where $S=I_{K\alpha }/C$ is the sensitivity (counts/s per concentration unit), t=100 s, and $k_{\mathrm{cal}}=1.026$ is the dimensionless calibration factor.

## 3. Simulation Results and DCC Design Optimization

### 3.1. Simulation Environment and Calibration Basis

The experimental reference data were obtained from internal laboratory measurements carried out in 2023–2024 on two vacuum prototype platforms, the Prototype Test System (PTS) and Super-X. The hardware stack included X-ray tubes with Cr, Ag, and Mo anodes; Ge and LiF crystals; a DCC optic holder; a vacuum pump; and a silicon drift detector (SDD) [34]. The baseline simulation conditions were kept consistent with the code: tube voltage 50 kV, tube current 40 mA, counting time 100 s, a silicate sample matrix, an SDD detector with an effective area of 30 mm^2^, and source-to-crystal and crystal-to-sample distances of 150 mm. The vacuum measurements were normally performed below 500 Pa, with high-quality runs reaching 100 Pa or below to reduce air attenuation. It is important to distinguish the parameter sources used in the model: the tube voltage, tube current, counting time, distances, detector type, and part of the geometry are experimental settings or instrument test conditions; the integrated reflectivity, rocking curve width, Ag/Cr flux ratio, voltage–intensity trend, and bending uniformity are measured laboratory anchors; and the characteristic line energies, fluorescence yields, absorption edges, mass attenuation coefficients, crystal *d*-spacings, densities, Darwin widths, and fundamental physical constants are taken from public databases or standard physical data.

The 50 kV tube voltage, 40 mA tube current, silicate matrix, and 30 mm^2^ SDD are the experimental platform conditions inherited from the reference measurements; 50 kV is an experimental reference rather than an optimized value (the scan shows a shallow minimum near 60 kV). The 100 s counting time is a fixed comparison time, and the p=q=150 mm distances denote a common benchmark geometry treated as variables in the scans below. The silicate matrix is a single representative matrix, so absolute LOD values do not transfer directly to other matrices.

The simulation input basis is separated into two levels. First, calculated tube spectra for Cr, Ag, and Mo anodes provide the energy-dependent excitation flux used by the diffraction and XRF forward models, with the Fe K-edge and Fe excitation window defining the relevant spectral region. Second, reference anchors define the confidence of the calibration constraints. Anchors with explicit uncertainty estimates provide the main quantitative constraints, whereas the LiF(220) integrated reflectivity is treated as a nominal auxiliary reference because no independent repeat-measurement uncertainty was reported.

Nine reference values (Table 1) constrain the three main sources of model uncertainty—crystal efficiency, tube spectrum, and bending uniformity—and map onto specific model components: integrated reflectivities constrain the absolute diffraction scale, rocking and energy widths constrain the angular and spectral acceptance, the Ag/Cr ratio and voltage trend constrain the source term, and the R85 uniformity measurement constrains the bending efficiency penalty. Because only a single bending uniformity datum ($R_{m}=85$ mm) is available, this anchor constrains the meridional efficiency model at one radius.

The ED-XRF, flat-crystal, and single-bend entries are included only to provide context under comparable laboratory assumptions; they are not part of the formal calibration or self-consistency dataset analyzed below.

The global calibration factor $k_{\mathrm{cal}}$ is obtained by minimizing the mean relative error (MRE) between model predictions and a synthetic set of comparison values constructed as follows: For each of the 13 DCC configurations, five replicate LOD values are generated by applying multiplicative perturbations drawn from the range [0.901, 1.159], together with replicate noise, to emulate the fabrication tolerances and alignment variability encountered in laboratory practice. These 13 configurations are defined independently of the nine reference anchors in Table 1: no anchor value is reused as a comparison target, ensuring that calibration and self-consistency assessment draw on non-overlapping information. The configuration set below defines the design space used throughout the following comparisons. All cases share the baseline above and use Fe Kα as the analyte line and 17.479 keV as the nominal monochromator design energy. The key varied parameters are as follows:

- C01: Ge(111) baseline with Ag anode, $R_{m}=R_{s}=150$ mm, p=q=150 mm, and 30×20 mm crystal size.
- C02: LiF(200) baseline under the same geometry and Ag-anode conditions.
- C03: LiF(220) baseline under the same geometry and Ag-anode conditions.
- C04: Ge(111) case with tighter meridional bending, $R_{m}=85$ mm and $R_{s}=150$ mm.
- C05: Ge(111) case with tighter sagittal bending, $R_{m}=150$ mm and $R_{s}=85$ mm.
- C06: Ge(111) asymmetric curvature case with $R_{m}=200$ mm and $R_{s}=100$ mm.
- C07: Ge(111) weak meridional bending case with $R_{m}=300$ mm and $R_{s}=150$ mm.
- C08: Ge(111) short-distance case with p=q=100 mm.
- C09: Ge(111) long-distance case with p=q=200 mm.
- C10: Ge(111) smaller-crystal case with 20×15 mm active area.
- C11: Ge(111) larger-crystal case with 40×30 mm active area.
- C12: Ge(111) source material case using a Cr anode.
- C13: Ge(111) source material case using a Mo anode.

Minimizing the MRE over the full 5×13 synthetic dataset yields $k_{\mathrm{cal}}=1.026$, indicating that the model requires only a 2.6% global scale correction to reproduce the center of the perturbed distribution.

Figure 3 shows predicted versus synthetic comparison LOD values for all 13 DCC configurations.

All 13 configurations fall within the ±20% band (10 of 13 within ±10%), with r=0.9833 and MRE =7.66% over a 6.9× dynamic range (LiF(220): 1.253ppm to C07: 8.593ppm). The residuals do not concentrate in any single parameter family, indicating that the model captures the leading-order trends shared across the explored axes rather than one favored baseline. This supports comparative screening within the explored design space but does not constitute independent experimental validation of absolute LOD values.

### 3.2. Geometric Parameter Effects

Figure 2 shows the two-dimensional focusing mechanism of a DCC optic and compares it with a single-bend geometry: the meridional radius $R_{m}$ controls dispersion-plane focusing (panel a); the sagittal radius $R_{s}$ adds transverse focusing (panel b); and the DCC solid-angle gain rises with the sagittal aperture *W*, while the single-bend reference stays nearly independent of *W* (panel c). The elongated intensity map (panel d) indicates that the compact geometry forms an anisotropic focal footprint rather than a circular spot.

In the meridional plane, the Rowland circle condition $R_{m}^{\mathrm{opt}}=2p/\sin\theta_{B}$ yields large ideal meridional radii for both Ge(111) and LiF(200) at p=150 mm, far exceeding the compact laboratory scale. Figure 2, however, intentionally displays the more compact anchored geometry used for the benchmark illustrations, with $R_{m}=R_{s}=85$ mm, such that the ray paths and anisotropic focal spot can be visualized on the laboratory scale of interest. For bulk XRF, this places a requirement on sample lateral uniformity and on matching the illuminated footprint to the sample area, whereas for spatial mapping or highly localized excitation, this would be undesirable. In such applications, larger meridional radii, tighter aperturing, or a different acceptance trade-off would be required to reduce the extreme aspect ratio of the focal footprint.

Figure 4 shows how *p* and *q* affect the Fe detection limit: ${\mathrm{LOD}}_{\mathrm{Fe}}$ decreases with *p* under q=p (panel a) and with the distance ratio q/p (panel b), and the low-${\mathrm{LOD}}_{\mathrm{Fe}}$ region of the (p,q) map (panel c) shifts toward larger *p* and *q*.

The ${\mathrm{LOD}}_{\mathrm{Fe}}$ decreases with increasing *p*: *p* enters through two competing terms—the collection solid angle ($\Omega_{\mathrm{DCC}}\propto 1/p^{2}$, suppressing flux at large *p*) and the meridional curvature-matching reference ($R_{m}^{\mathrm{ref}}\propto p$, shifting the focusing efficiency window toward the buildable radius)—and the latter dominates over the sampled range. At p=150 mm, the ${\mathrm{LOD}}_{\mathrm{Fe}}$ also decreases monotonically over the scanned q/p interval, as increasing *q* improves the sagittal focusing condition and the sample-coupling efficiency $\eta_{s}$. The lowest displayed region lies on the high-*p* side of the map rather than the high-${\mathrm{LOD}}_{\mathrm{Fe}}$ lower-left corner, so no interior optical optimum exists and distance selection is governed by instrument layout constraints.

Figure 5 evaluates the influence of crystal length *L* and width *W* regarding the collection efficiency and LOD: increasing *L* raises the solid angle Ω and reduces ${\mathrm{LOD}}_{\mathrm{Fe}}$ (panel a), *W* acts analogously (panel b), and panel (c) maps Ω over the (W,L) plane.

The ${\mathrm{LOD}}_{\mathrm{Fe}}$ decreases with increasing *L*: the solid-angle gain Ω∝L dominates the Johann aberration penalty ($\delta_{\mathrm{Johann}}\propto L^{2}/R_{m}^{2}$, which broadens the rocking curve and raises $\beta_{J}$) over the scanned 15–50 mm interval. For *W*, ${\mathrm{LOD}}_{\mathrm{Fe}}$ decreases monotonically with no aberration penalty, so no interior optimum is observed. The recommended (L,W)=(30,20) mm balances aberration growth and fabrication constraints against the monotonic optical gain.

### 3.3. Crystal Material and Mosaicity Effects

Figure 6 compares the diffraction responses of Ge(111), LiF(200), and LiF(220) at 17.48 keV. The normalized rocking curves (panel a) show Ge(111) as the narrowest profile—better angular and energy selectivity—and the broader LiF curves as showing larger angular acceptance; because the curves are normalized, absolute reflectivity is compared separately through $R_{\mathrm{int}}$ (panel b). LiF(200) and LiF(220) achieve higher $R_{\mathrm{int}}$ than Ge(111) (greater diffracted flux collection), while Ge(111) has the narrowest energy bandwidth (superior spectral discrimination at the cost of lower throughput). The key parameters are summarized in Table 2.

Relative to Ge(111), LiF(220) provides 3.09 times the integrated reflectivity with 1.70 times the bandwidth; compared with LiF(200), it provides 13.3% higher $R_{\mathrm{int}}$ and 44.3% narrower ΔE. LiF(220) therefore retains the largest reflectivity-related signal gain while imposing less bandwidth-related background than LiF(200), yielding the lowest benchmark LOD.

Figure 7 shows how the mosaicity η changes the DCC crystal response: increasing η broadens the rocking curve and lowers its peak (panel a); reduces peak reflectivity while the integrated response remains useful (panel b); and monotonically increases the energy bandwidth ΔE (panel c), degrading the energy resolution.

As η increases, the crystallite orientation distribution broadens and a wider range of incident angles satisfies the local Bragg condition, allowing diffraction over a wider angular range. Through the Bragg relation, this angular broadening produces a wider energy response and distributes the reflected intensity over a broader interval, thereby reducing the peak amplitude while the effective width $\Delta \theta_{\mathrm{eff}}$ and accepted background grow. The increased acceptance may therefore admit more background, so excessive mosaicity can degrade the peak-to-background ratio and LOD despite improving the angular acceptance. The reference point in Figure 7b,c is well-reproduced. Within the sampled mosaicity grid, $\eta =0.15^{\circ }$ represents an approximate engineering upper bound for the present configuration: in the simulated range the acceptance gain remains competitive up to approximately $\eta =0.15^{\circ }$, whereas beyond this value the bandwidth-related background and peak reflectivity loss increasingly offset the throughput benefit. This recommended condition is therefore based on the modeled LOD trade-off rather than on a universal physical discontinuity. Its precise location depends on the crystal material, reflection plane, photon energy, crystal thickness, source divergence, detector response, and background conditions, and is not inferred solely from the $0.10^{\circ }$ calibration anchor; a finer scan around $\eta =0.15^{\circ }$, together with independent rocking curve and LOD measurements, would be required to determine a more precise configuration-specific threshold.

### 3.4. Source Material and Operating Power Effects

Figure 8 places the source comparison in spectral context. At the nominal DCC energy of 17.479 keV, the Ag and Cr anodes contribute mainly through their bremsstrahlung continua because their Kα lines do not coincide with this energy, whereas the Mo Kα line lies near 17.479 keV and can contribute within the modeled monochromator response. The shaded region above the Fe K-edge (7.112 keV) marks the Fe K-shell excitation range and is not the DCC transmission window.

Figure 9 shows how the source material and tube voltage affect the modeled Fe detection limit for the Ge(111) baseline: anode material (panel a), LOD normalized to the Ag case versus modeled relative source flux (panel b), and tube voltage (panel c).

Ag gives the highest modeled output flux ($4.810\times 10^{10}$ ph/s), followed by Mo ($4.357\times 10^{10}$) and Cr ($1.826\times 10^{10}$). The LOD ranking is set by the combined output flux and crystal peak-to-background ratio rather than the presence of a characteristic line, so Ag yields the most favorable flux–background balance and the lowest LOD. Because the modeled Ag/Cr source flux ratio is below the measured anchor (Section 2.2), the modeled Ag advantage over Cr is conservative.

### 3.5. Curvature Radius Effects in Hyperbolic DCC Design

Figure 10 reports the coupled optimization of the two hyperbolic bending radii: ${\mathrm{LOD}}_{\mathrm{Fe}}$ and normalized integrated flux versus $R_{m}$ (panel a), ${\mathrm{LOD}}_{\mathrm{Fe}}$ and sagittal focal spot size versus $R_{s}$ (panel b), and two-dimensional ${\mathrm{LOD}}_{\mathrm{Fe}}$ and integrated flux maps over $\left(R_{s},R_{m}\right)$ (panels c,d).

The 1-D $R_{m}$ scan ($R_{s}=20$ mm fixed) gives its lowest sampled value at $R_{m}=85$ mm, coinciding with the calibration anchor and far from the theoretical Rowland radius of 2.76 m, so it may partly reflect the calibration. The 1-D $R_{s}$ scan ($R_{m}=75$ mm fixed) gives its lowest sampled value at $R_{s}=20$ mm, close to the theoretical 16.3 mm (the small difference reflects the discrete grid and additional focal spot terms). The joint minimum at $\left(R_{m},R_{s}\right)=\left(75,20\right)$ mm is therefore a conditional sampled minimum.

### 3.6. Integrated DCC Design Optimization

To clarify the mechanisms underlying the parameter–LOD trends, Equations (18)–(20) give, for a fixed analyte, matrix, detector, and counting time,

$$\mathrm{LOD}\propto \sqrt{\frac{\beta_{J}}{\Phi_{\mathrm{out}}\;{\left(P/B\right)}_{\mathrm{crystal}}}},\;\Phi_{\mathrm{out}}\propto \Phi_{\mathrm{in}}\;R_{\mathrm{int}}\;\Omega_{\mathrm{DCC}}\;\eta_{m}\;\eta_{s},$$

with fixed transmission and calibration factors omitted for clarity. Thus, $R_{m}$ affects LOD through $\eta_{m}$ and $\delta_{\mathrm{Johann}}\to \Delta \theta_{\mathrm{eff}}\to \beta_{J}$; $R_{s}$ acts through $f_{\mathrm{spot}}\to \eta_{s}$; p/q acts through collection and focusing; L/W increases $\Omega_{\mathrm{DCC}}$, while *L* also increases Johann broadening; and crystal material or mosaicity acts through $R_{\mathrm{int}}$, $\Delta \theta_{\mathrm{eff}}$, and ${\left(P/B\right)}_{\mathrm{crystal}}$. These pathways separate the throughput, focusing, and background contributions underlying the observed LOD trends.

Figure 11 presents the ranked LOD values for the top configurations together with a multi-parameter comparison.

Panel (a) ranks the explicitly evaluated configurations by Fe Kα LOD (I–VIII = C03, C02, C05, C11, C04, C09, C01, C06). Panel (b) places the same configurations in flux–LOD space colored by engineering score. Panel (c) instead plots sampled radii from the curvature scans ($R_{m}$ at $R_{s}=20$ mm, $R_{s}$ at $R_{m}=75$ mm) and is not another top-configuration ranking.

For curvature radii above approximately 400 mm, the modeled LOD changes only weakly within the sampled range, indicating an apparent rather than strict saturation. As $R_{m}$ increases, the Johann broadening decreases as $\delta_{\mathrm{Johann}}\propto R_{m}^{-2}$; once it becomes small relative to the mosaicity and Darwin width contributions, further increases in radius have little influence on $\Delta \theta_{\mathrm{eff}}$ and $\beta_{J}$. The LOD is then mainly governed by the source flux, integrated reflectivity, solid angle, and intrinsic diffraction width. Therefore, radii beyond this range provide limited analytical improvement while potentially increasing the system footprint and alignment requirements. This plateau is conditional on the present model and scan range rather than a universal saturation radius.

Panel (d) plots normalized penalty polylines for the top five configurations (LiF(220), LiF(200), C05, C11, C04); a low, flat line indicates a configuration that is optically strong and balanced across background and implementation metrics, so the lowest-LOD configuration and the most attractive engineering choice need not coincide.

Two conclusions follow: First, LiF(220) achieves the lowest LOD among the explicitly evaluated configurations while maintaining an acceptable engineering score. Second, the Ge(111) curvature scan minimum is a conditional sampled minimum not directly comparable with the ranked configurations, which use their own anchored parameter sets.

Table 3 summarizes the recommended engineering parameters.

The comparison in Table 3 is a decision aid rather than a winner-take-all ranking: the Ge(111) curvature scan minimum shows what is achievable by refining geometric parameters within one crystal family, while the LiF(220) baseline is more fabrication-tolerant under shared geometry.

### 3.7. Model Scope and Limitations

Several limitations define the scope of this framework. The diffraction module is a reduced-order surrogate, and the focusing factors $\eta_{m}$ and $\eta_{s}$ and the background penalty $\beta_{J}$ are empirical or phenomenological, with the curvature response constrained by a single bending uniformity anchor at $R_{m}=85$ mm. In particular, $\eta_{m}$ has not been validated by multi-radius bending efficiency measurements, and $\eta_{s}$ has not been quantitatively compared with independent ray-tracing software or multi-radius focal spot experiments. The resulting curvature trends and their lowest values therefore apply only to the present model, its calibration range, and the scan grid, and should be read as conditional screening results rather than universal or experimentally validated optima. Likewise, the 13-configuration dataset is a synthetic perturbation-based internal self-consistency check rather than an independent experimental validation. The framework is therefore intended for comparative screening and design trend analysis within the explored conditions; absolute LOD prediction requires an independent measurement campaign.

## 4. Conclusions

The calibrated framework integrates tube spectrum modeling, a Takagi–Taupin-informed diffraction module, two-dimensional DCC focusing geometry, and an XRF forward model into a unified source-to-LOD chain. Among the 13 shared-geometry configurations, LiF(220) achieves the lowest LOD at 1.253ppm ($R_{m}=R_{s}=150$ mm, p=q=150 mm), combining high integrated reflectivity with a favorable throughput–resolution compromise. A conditional Ge(111) curvature scan reaches a sampled minimum of 1.094ppm within the specified curvature scan at $\left(R_{m},R_{s}\right)=\left(75,20\right)$ mm, isolating radius sensitivity rather than a direct crystal-to-crystal comparison; Ag yields the lowest LOD among the three anodes. Absolute LOD prediction still requires an independent measurement campaign.

Within the explored design region, curvature parameters provide the largest local geometric leverage, followed by source distance and crystal dimensions, with anode selection producing a more modest effect. Three coupling relationships govern the design space: $R_{m}$ and *L* through Johann aberration, $R_{s}$ and *q* through the sagittal focusing condition, and crystal material and η through $R_{\mathrm{int}}$ and ΔE. A practical optimization sequence is as follows: crystal material → fix p/q→ optimize $R_{m}$→ optimize L/W → fine-tune $R_{s}$. The DCC solid-angle gain over single-bend optics, $G=W/(L\;\Delta \theta_{\mathrm{eff}})$, grows linearly with *W*, though fabrication complexity—simultaneous control of two curvature radii, slope error, and bending uniformity—remains the principal practical limitation. The framework transfers to other crystals, sources, and target elements in the hard X-ray range.

## Figures and Tables

**Figure 1:**
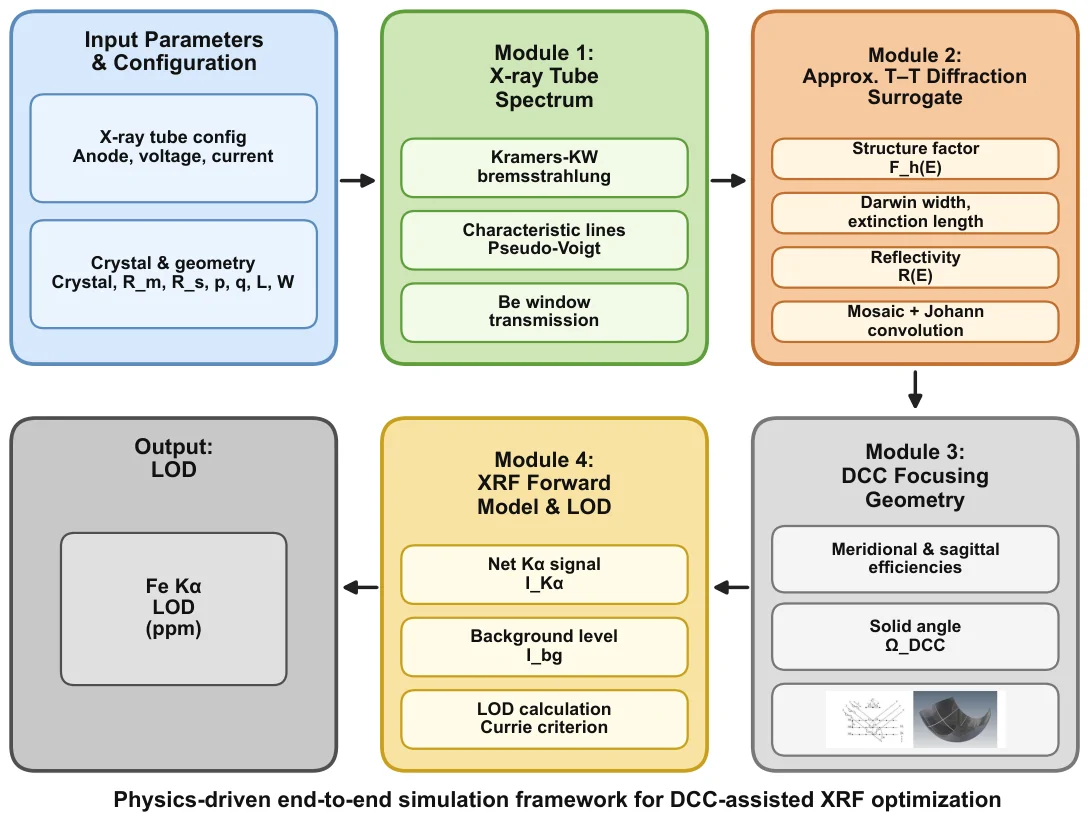
End-to-end simulation framework for DCC-based XRF optimization. The workflow links the source generation, crystal diffraction, focusing geometry, and XRF forward modeling.

**Figure 2:**
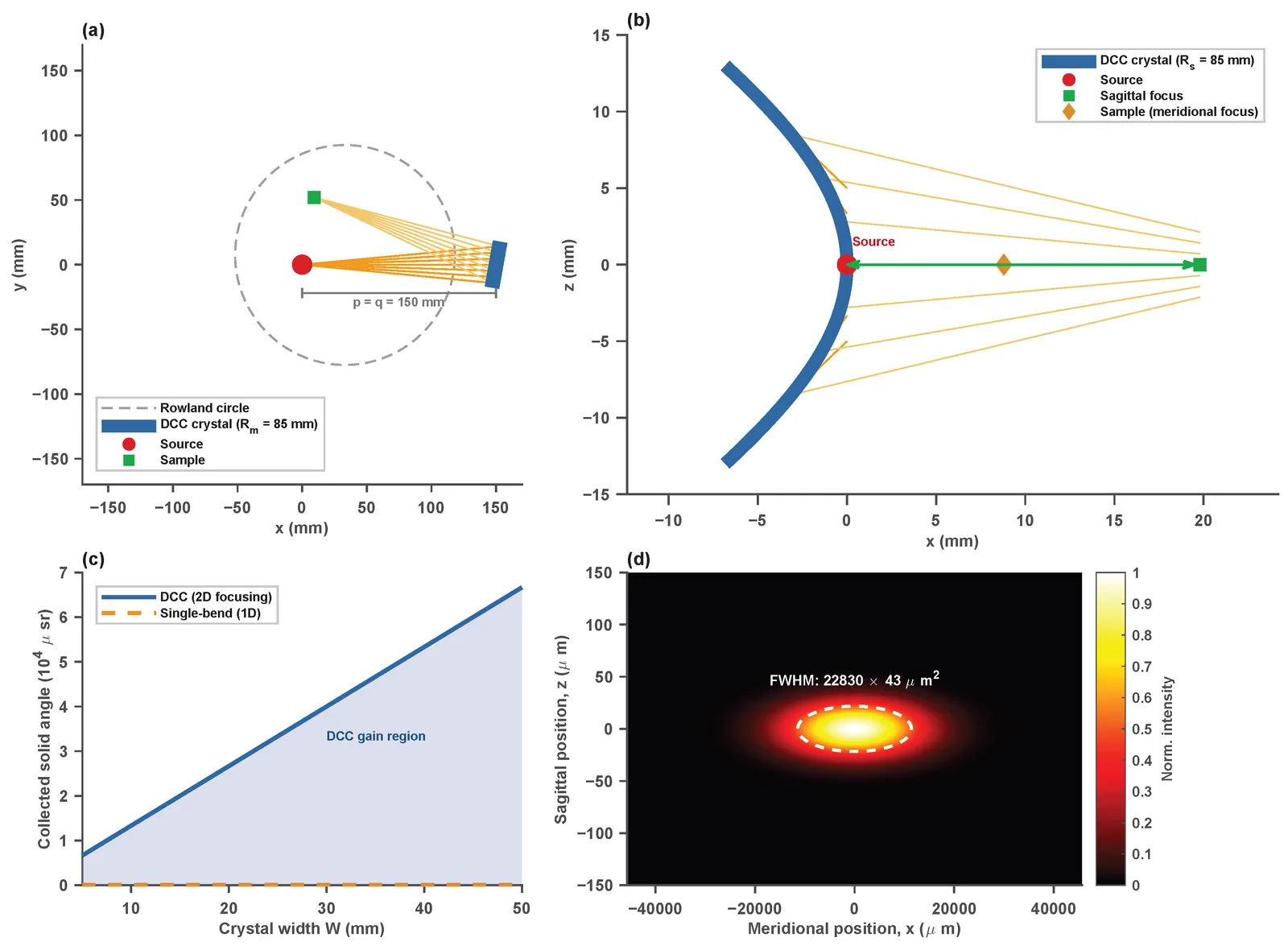
Representative model-generated DCC focusing geometry. (**a**) Meridional ray view. (**b**) Sagittal focusing view. (**c**) Solid-angle comparison with single-bend optics. (**d**) Modeled focal spot map.

**Figure 3:**
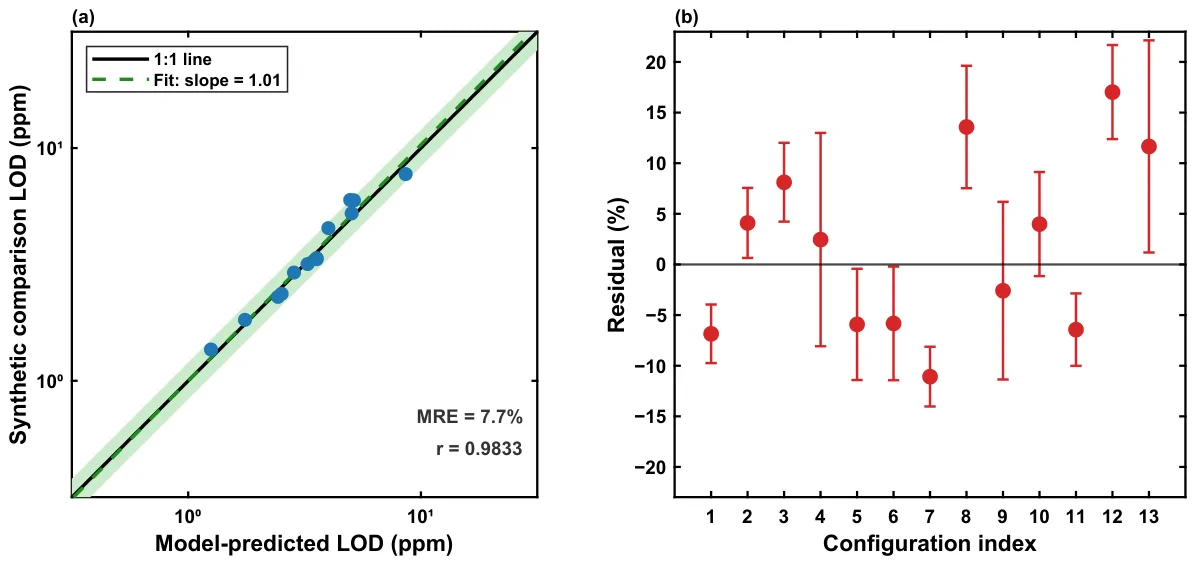
Synthetic internal self-consistency check. (**a**) Predicted versus synthetic comparison LOD with a ±20% band. (**b**) Relative residuals for the 13 configurations; error bars denote synthetic replicate standard deviations. Blue circles represent the 13 DCC configurations, and the green shaded region denotes the ±20% agreement band around the 1:1 line.

**Figure 4:**
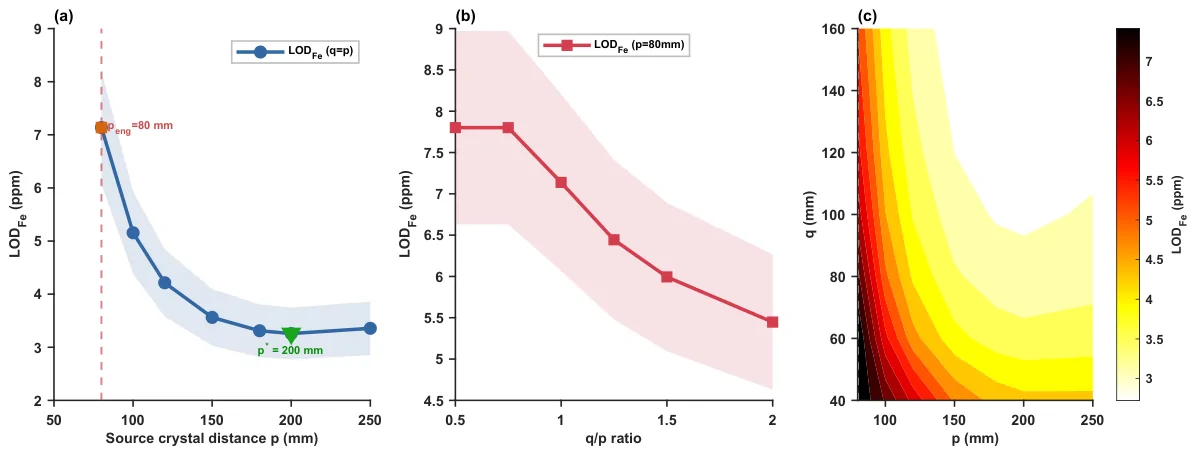
Distance–parameter trends for the Ge(111) DCC baseline. (**a**) ${\mathrm{LOD}}_{\mathrm{Fe}}$ versus source-to-crystal distance *p*. (**b**) ${\mathrm{LOD}}_{\mathrm{Fe}}$ versus distance ratio q/p. (**c**) Two-dimensional ${\mathrm{LOD}}_{\mathrm{Fe}}$ map in (p,q) space. In panel (**a**), the shaded area is a visual fill beneath the modeled LOD curve and does not represent an uncertainty interval; the dashed vertical line marks the lowest sampled LOD at p=200 mm.

**Figure 5:**
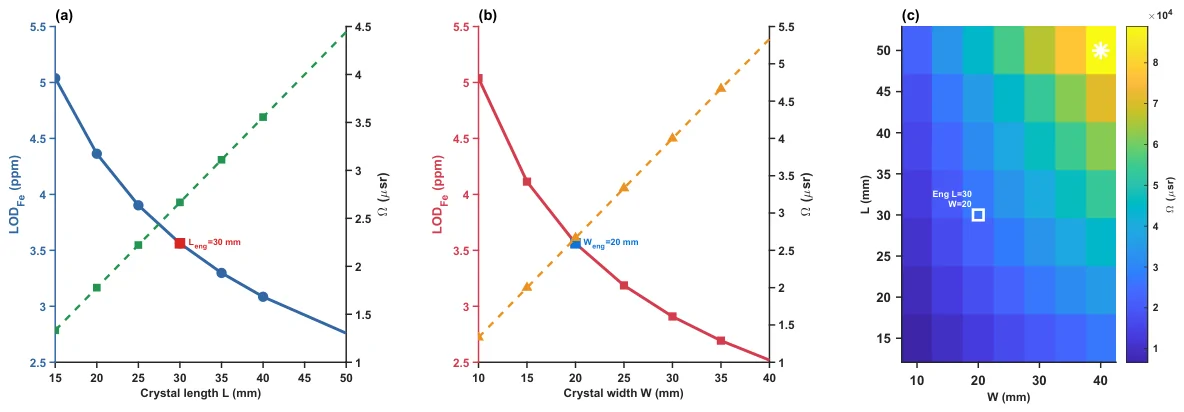
Crystal size trends for the Ge(111) DCC baseline. (**a**) ${\mathrm{LOD}}_{\mathrm{Fe}}$ and solid angle versus crystal length *L*. (**b**) ${\mathrm{LOD}}_{\mathrm{Fe}}$ and solid angle versus crystal width *W*. (**c**) Two-dimensional solid-angle map in (L,W) space. In panels (**a**) and (**b**), the LOD curves correspond to the left vertical axes, whereas the solid-angle curves correspond to the right vertical axes; the vertical reference lines mark the engineering dimensions L=30 mm and W=20 mm, respectively.

**Figure 6:**
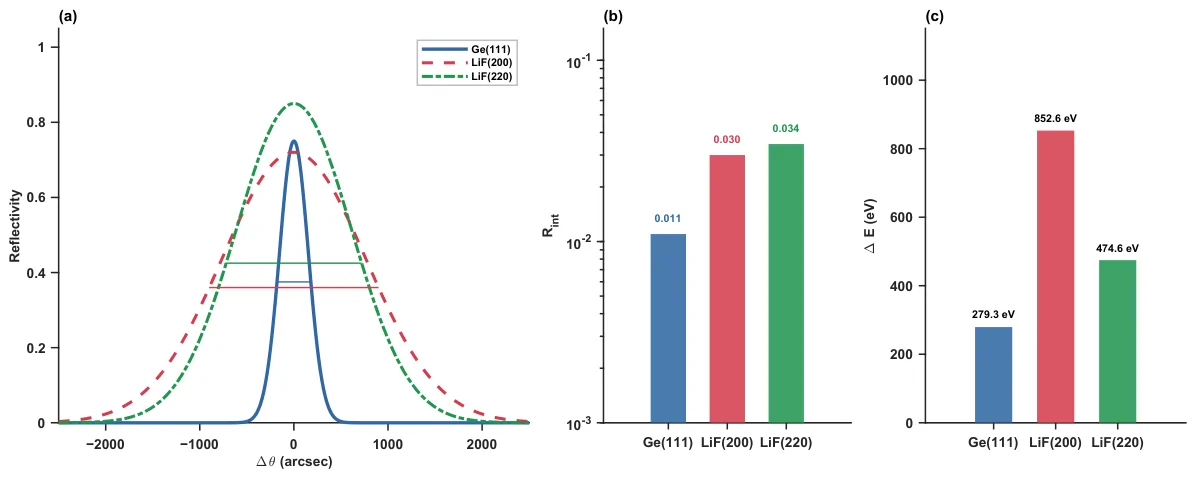
Diffraction–property comparison of the candidate DCC crystals at 17.48 keV. (**a**) Normalized rocking curves. (**b**) Relative reflectivity. (**c**) Relative energy resolution. In panel (**a**), the solid horizontal line indicates the unit normalized-reflectivity level used to compare the rocking-curve profiles.

**Figure 7:**
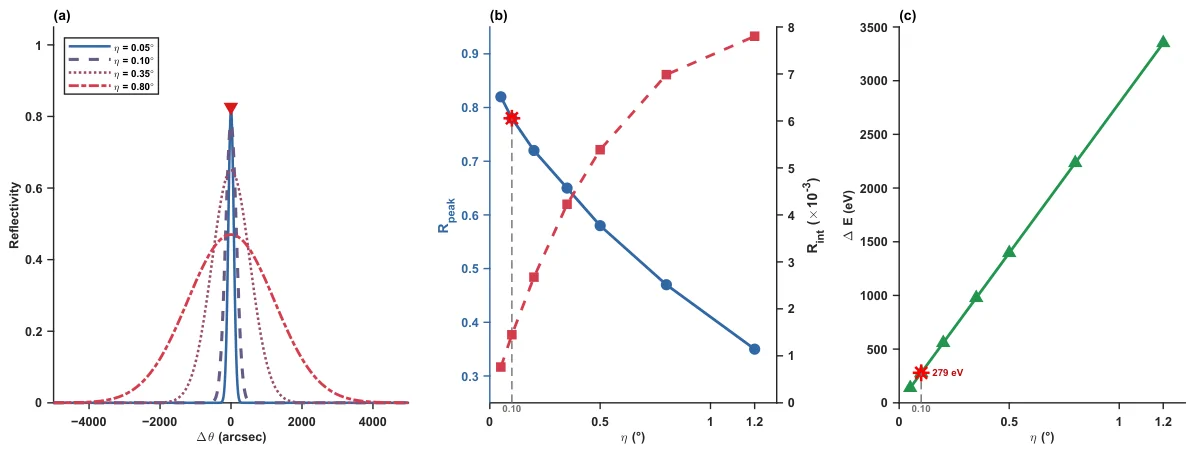
Influence of mosaicity η on DCC crystal response. (**a**) Rocking curve evolution with increasing η. (**b**) Reflectivity trend. (**c**) Energy resolution trend. In panel (**b**), the peak-reflectivity and integrated-reflectivity curves correspond to the left and right vertical axes, respectively; in panel (**c**), the curve represents the modeled energy bandwidth. The reference markers at $\eta =0.10^{\circ }$ indicate the calibration condition, including the reference bandwidth of 279 eV.

**Figure 8:**
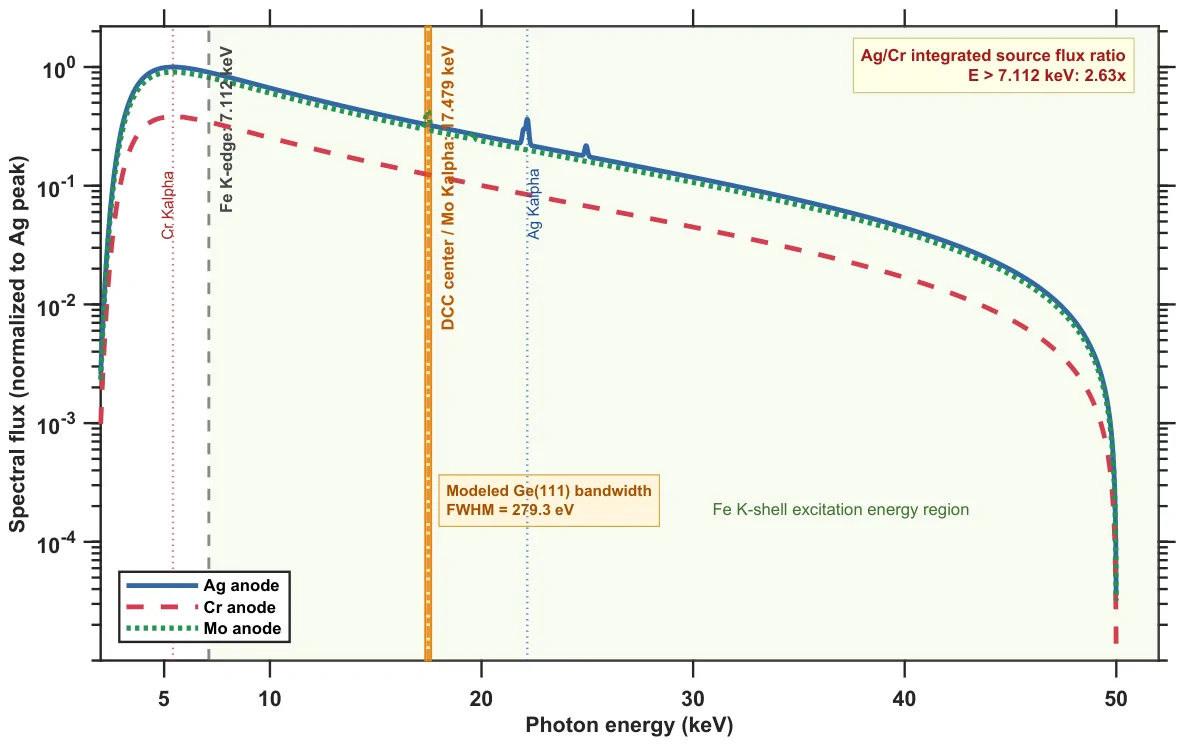
Simulated Ag, Cr, and Mo X-ray tube source spectra normalized to the Ag spectral peak, with the Fe K-edge (7.112 keV), the nominal DCC energy (17.479 keV), and the modeled Ge(111) FWHM bandwidth (279.3 eV) indicated; the shaded region marks the Fe K-shell excitation range.

**Figure 9:**
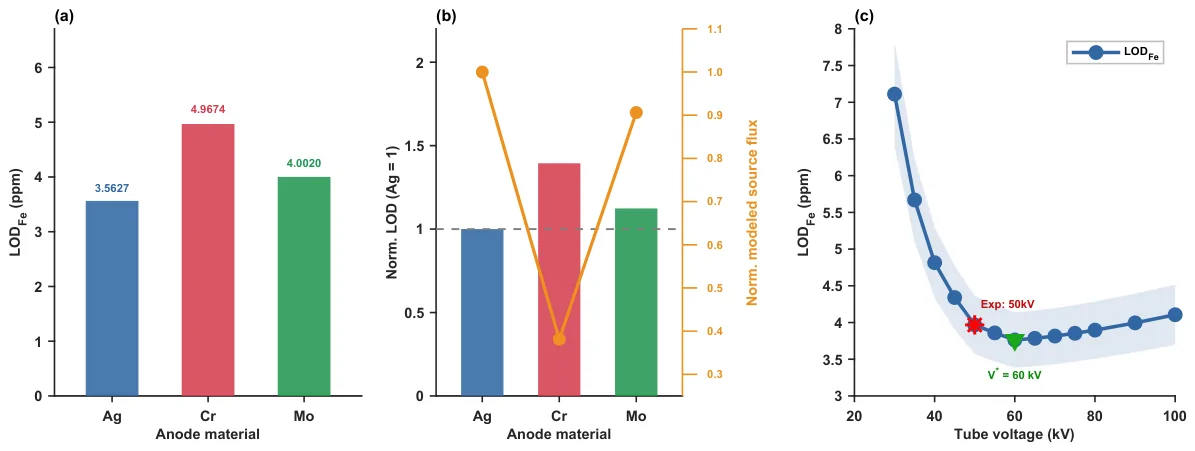
X-ray tube source material and operating power trends for the Ge(111) DCC baseline. (**a**) ${\mathrm{LOD}}_{\mathrm{Fe}}$ versus anode material. (**b**) Normalized ${\mathrm{LOD}}_{\mathrm{Fe}}$ and continuum comparison. (**c**) ${\mathrm{LOD}}_{\mathrm{Fe}}$ versus tube voltage. In panel (**c**), the blue shaded area is a visual fill beneath the modeled LOD–voltage curve and does not represent an uncertainty interval; the vertical markers identify the sampled minimum at 60 kV and the experimental reference condition at 50 kV.

**Figure 10:**
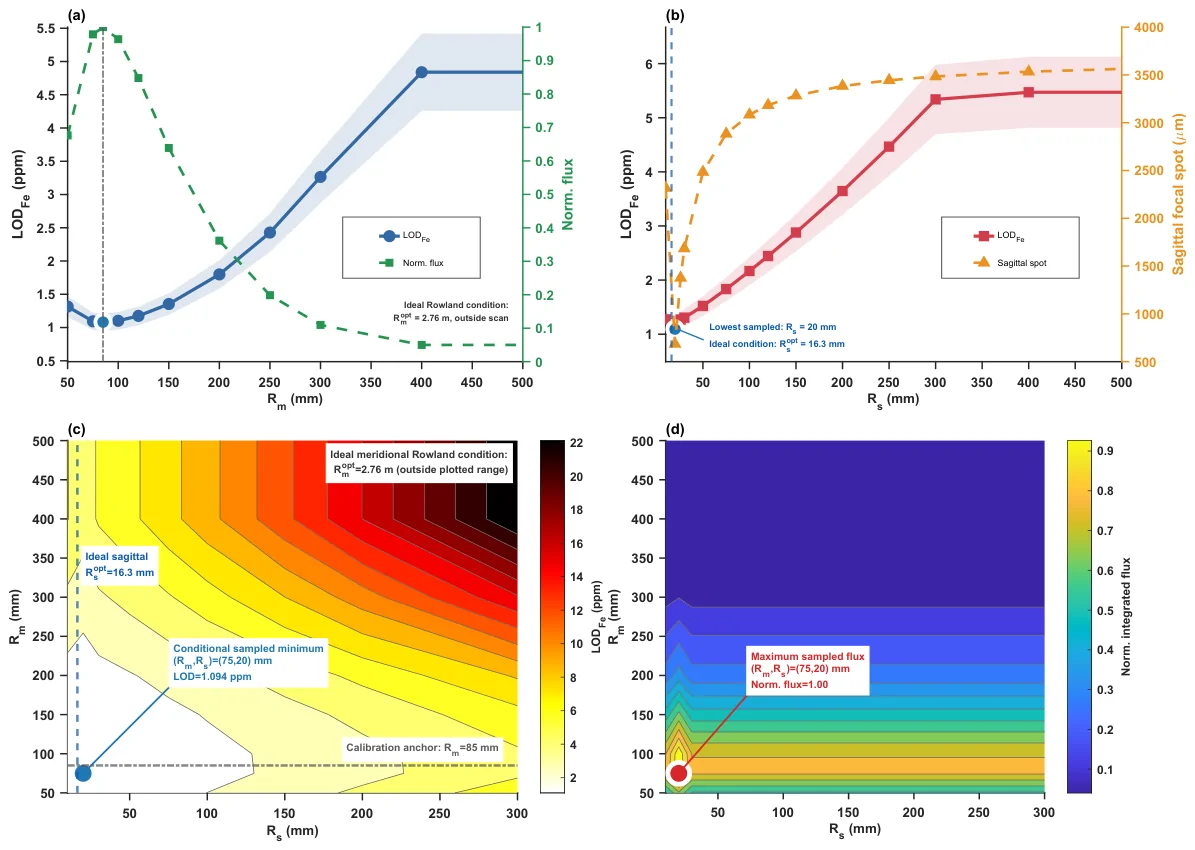
Conditional curvature scan for the Ge(111) DCC baseline. (**a**) ${\mathrm{LOD}}_{\mathrm{Fe}}$ and normalized flux versus $R_{m}$. (**b**) ${\mathrm{LOD}}_{\mathrm{Fe}}$ and sagittal focal spot size versus $R_{s}$. (**c**) Two-dimensional ${\mathrm{LOD}}_{\mathrm{Fe}}$ map with the conditional sampled minimum and calibration anchor. (**d**) Normalized integrated flux map. In panel (**a**), the red and blue shaded fills accompany the modeled LOD and normalized-flux curves, respectively; in panel (**b**), they accompany the modeled LOD and sagittal-focal-spot curves, respectively. These colored fills are used only to distinguish the paired quantities and do not represent confidence or uncertainty intervals. In panels (**c**,**d**), the colors represent the LOD and normalized integrated-flux values specified by the corresponding color bars.

**Figure 11:**
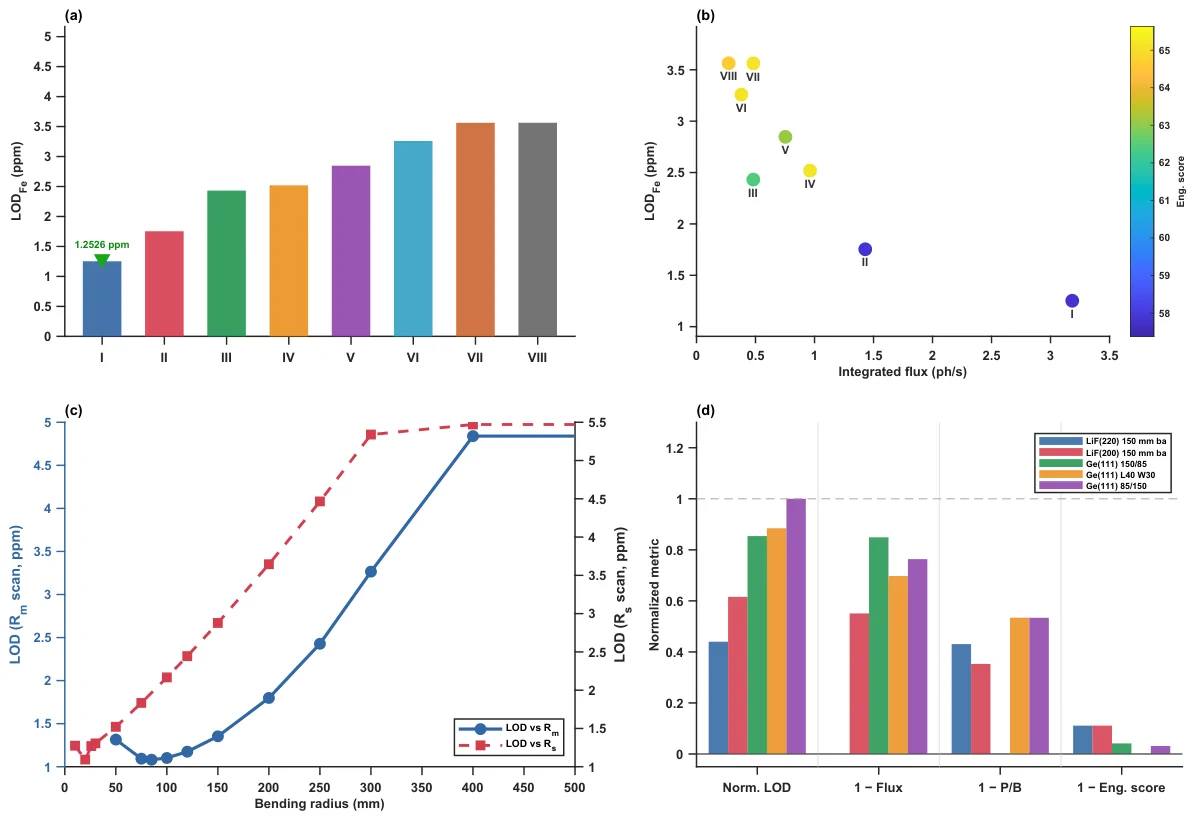
Summary of representative DCC configurations for the Fe Kα benchmark. (**a**) LOD ranking of the eight best configurations. (**b**) Flux–LOD trade-off colored by engineering score. (**c**) Curvature scan response for $R_{m}$ and $R_{s}$. (**d**) Normalized multi-metric penalty profiles for the top five configurations.

**Table 1:** Reference data used for model calibration and subsequent self-consistency analysis.

Reference Datum	Value	Constrained Parameter
Ge(111) DCC $R_{\mathrm{int}}$	1.1%±0.1%	T-T absolute efficiency
LiF(200) DCC $R_{\mathrm{int}}$	3.0%±0.5%	T-T absolute efficiency
LiF(220) DCC $R_{\mathrm{int}}$	3.4% (nominal)	Auxiliary T-T reference
Ge(111) rocking FWHM	360 arcsec	Mosaicity η
Ge(111) ΔE	279 eV	Darwin width
LiF(200) ΔE	853 eV	Mosaic broadening
Ag/Cr flux ratio	3.20±0.30	Kramers coefficient
Experimental operating voltage	50 kV	Voltage–flux relation
DCC bending uniformity	78% ($R_{m}=85$ mm)	Bending efficiency factor

**Table 2:** Diffraction parameters of DCC candidate crystals at 17.48 keV. $R_{\mathrm{int}}$ values from reference data; ΔE from Figure 6c.

Crystal	*d* (Å)	$\theta_{B}$	$R_{\mathrm{int}}$ (DCC)	ΔE (eV)
Ge(111)	3.266	$6.24^{\circ }$	1.1%	279.3
LiF(200)	2.014	$10.14^{\circ }$	3.0%	852.6
LiF(220)	1.424	$14.4^{\circ }$	3.4%	474.6

**Table 3:** Illustrative comparison of two non-equivalent reference cases: Ge(111) curvature scan minimum and engineering LiF(220) baseline (Fe Kα benchmark, the two columns compile results from different scans and reference configurations and should not be interpreted as either a single simultaneously realized design point or a single common optimum, $k_{\mathrm{cal}}=1.026$).

Parameter	Curvature Scan Minimum	Engineering Recommendation	Constraint
Crystal	Ge(111)	LiF(220)	Reference case
$R_{m}$ (mm)	75	150	Bending uniformity anchor
$R_{s}$ (mm)	20	150	Fabrication precision
*p* (mm)	200	150	Mechanical clearance
*q* (mm)	400	150	Detector layout
*L* (mm)	50 (scan edge)	30	Scan upper bound
*W* (mm)	40 (scan edge)	20	Scan upper bound
${\mathrm{LOD}}_{\mathrm{Fe}}$ (ppm)	1.094	1.253	—

## Data Availability

The datasets presented in this article are not readily available because they contain proprietary industrial data from an internal company production process and are subject to confidentiality restrictions. Requests to access the datasets should be directed to the corresponding author.
